# Treatment fidelity in the Camden Weight Loss (CAMWEL) intervention assessed from recordings of advisor-participant consultations

**DOI:** 10.1186/s40608-018-0203-7

**Published:** 2018-09-10

**Authors:** Lorraine M Noble, Emma Godfrey, Liane Al-Baba, Gabriella Baez, Nicki Thorogood, Kiran Nanchahal

**Affiliations:** 1 UCL Medical School, University College London, Royal Free Hospital, Rowland Hill Street, NW3 2PF, London, UK; 2 Department of Psychology, Institute of Psychiatry, Psychology and Neuroscience, King’s College London, London, UK; 3 UCL Research Department of Epidemiology and Public Health, UCL, London, UK; 4 Department of Social and Environmental Health Research, London School of Hygiene and Tropical Medicine, London, UK

**Keywords:** Obesity, Overweight, Weight loss, Trial, Intervention, Fidelity, Motivational interviewing, Therapeutic alliance, Communication

## Abstract

**Background:**

Variations in the delivery of content and process can alter the effectiveness of complex interventions. This study examined the fidelity of a weight loss intervention (Camden Weight Loss) from recorded consultations by assessing advisors’ delivery of content, use of motivational interviewing approach and therapeutic alliance.

**Methods:**

A process evaluation was conducted of advisor-participant consultations in a 12-month randomised controlled trial of an intervention for adult volunteers with a body mass index categorised as overweight or obese. A convenience sample of 22 consultations (12% of 191 participants) recorded at the intervention mid-point were available for analysis. Consultations were independently rated by two observers independent of intervention or study delivery, using: a fidelity scale, the Motivational Interviewing Treatment Integrity Scale and the Primary Care Therapy Process Rating Scale. Raters were blind to participants’ responses to the intervention and weight outcomes. Half the participants (*N* = 11) achieved significant weight loss (≥ 5% of baseline weight).

**Results:**

A mean of 41% of prescribed content was delivered, with a range covered per session of 8–98%, falling below the 100% content expected per session. Tasks included most frequently were: taking weight and waist measurements (98%), scheduling next appointment (86%), review of general progress (85%) and reviewing weight change (84%). Individual items most frequently addressed were ‘giving encouragement’ and ‘showing appreciation of participant’s efforts’ (95 and 88% respectively). Consultation length (mean 19 min, range 9–30) was shorter than the 30-min allocation. Quantity of content correlated with consultation length (*p* < 0.01). Advisors’ use of motivational interviewing was rated at ‘beginner proficiency’ for Global Clinician Rating, Reflection to Question Ratio and Percent Open Questions. Therapeutic alliance scores were moderate. Affective aspects were rated highly (e.g. supportive encouragement, involvement and warmth).

**Conclusions:**

Intervention fidelity varied in both content and process, emphasising the importance of ongoing fidelity checks in a complex intervention. Advisors focused on certain practical aspects of the intervention and providing an encouraging interpersonal climate. This concurs with other research findings, which have revealed the value participants in a weight loss intervention place on an empathic advisor-participant relationship.

**Clinical trials registration:**

Registered with Clinicaltrials.gov, number NCT00891943, on 1 May 2009.

## Background

The increasing impact of obesity on health has caused international alarm [1, 2]. It is estimated that 2.8 million people die annually due to overweight or obesity [2]. In the UK, 65% of men, 58% of women and 33% of children aged 10–11 are overweight or obese [3]. Health problems related to obesity are estimated to cost the UK National Health Service £5 billion per year [4].

Guidance recommends multi-component weight management interventions focusing on dietary intake, physical activity and behaviour change, and that behaviour modification addresses: *‘problem solving; goal setting; how to carry out a particular task or activity; planning to provide social support or make changes to the social environment; self-monitoring of weight and behaviours that can affect weight; and feedback on performance’* [5]*.* Affective features of weight management interventions are also highlighted, emphasising empathy, support and encouragement, and a respectful and non-judgemental approach.

Guidance reflects the complexity of evidence about weight management and the theoretical basis for behaviour change [6–12]. Multi-component interventions are superior to single-component interventions and result in greater longer term weight loss than control conditions [7, 8]. However, intervention success varies, with variation not accounted for by participant characteristics or programme components (such as length, intensity or face-to-face contact). Long term weight loss remains a challenge [9, 12, 13].

Behaviour change interventions aim to encourage people to self-manage their weight in the long term. Motivational interviewing aims to support this by identifying and enhancing an individual’s own motivation and self-efficacy. The health professional employs an empathic, supportive and collaborative approach, emphasising the individual’s autonomy and encouraging the person to explore their own reasons for, and ambivalence about, changing the target behaviour [14].

Whilst motivational interviewing is effective in promoting behaviour change, many health professionals are ‘generalists’, using a variety of approaches rather than a single, ‘pure’ approach. ‘Motivational interviewing-style’ approaches, which employ some of the elements (such as empathy) without using the full range of techniques, have been investigated [15]. Weight management programmes including either pure or adapted forms of motivational interviewing improve outcomes relative to traditional behaviour change interventions or control conditions [16, 17]. However, in primary care consultations with patients who were overweight or obese, low levels of techniques consistent with a motivational interviewing approach were observed, specifically empathy and motivational interviewing ‘spirit’ [18].

The importance of the quality of the therapeutic relationship on outcomes of behaviour change interventions has also been recognised [19]. Therapeutic alliance includes affective aspects of the professional-patient relationship (such as empathy, rapport and warmth) and instrumental aspects (such as agreement on goals and tasks). Baldwin and colleagues highlighted the impact of therapeutic alliance in weight management outcomes, and noted importance of the professional’s contribution to developing this alliance [20].

Weight management interventions require professionals to skilfully select and deliver elements in line with evidence and an individual’s needs. The importance of initial training and continuing professional development has been highlighted [5]. Key features to promote fidelity (defined as the degree to which an intervention is delivered as intended) are staff training, supervision and an intervention manual [21]. Failure to implement the intervention as designed can result in a ‘Type III error’, where study results do not reflect the effects of the planned intervention [22]. Fidelity includes exposure, adherence to content and quality of delivery [21]. It is commonly assessed by trained observers, either live or from recordings [16, 21, 23]. For example, one study of fidelity in a behaviour change intervention for diabetes found that staff training improved motivational interviewing spirit [24].

In a 12-month weight loss intervention trial for obese and overweight volunteers, a third of the intervention group achieved clinically significant weight loss (5% or more of their baseline weight) [25]. The present study was designed to examine intervention fidelity, to explore whether differences in intervention delivery may have contributed to variability in intervention group outcome.

### Study aim

To investigate weight loss intervention fidelity through assessing the content and process of advisor-participant consultations. Specifically, to establish whether: (i) intervention topics and activities were delivered as intended; (ii) advisors’ consultation style was consistent with approaches to support lifestyle behaviour change, in particular, using a motivational interviewing approach and establishing a therapeutic alliance.

## Method

### Design

This was an independent evaluation examining fidelity of a multi-component weight loss intervention delivered by health advisors to participants with weight categorised as overweight and obese, in the intervention arm of a pragmatic randomised control trial in primary care. This was a descriptive, observational study, conducting a process evaluation using independent, blind ratings of recorded advisor-participant consultations. Fidelity of intervention content (scheduled topics and activities) and process (motivational interviewing and therapeutic alliance) were examined. Recordings were taken from the mid-point of the intervention to: (i) assess the therapeutic relationship that had developed, (ii) reduce the influence of participants’ and advisors’ awareness of intervention outcome (i.e. final weight change).

### Participants

Participants were adults attending the 12-month Camden Weight Loss programme during a two year research period. Consultations were recorded during a five month period. Written consent was obtained from all participants. Out of 191 participants who received the intervention, 104 audio or video-recordings were obtained for 42 participants during the recording period. Including only participants for whom final weight outcomes were available resulted in 34 participants. Of these, recordings from the three mid-intervention sessions were available for 27 participants. Due to problems with sound quality, recordings from five participants were excluded, resulting in a total sample of 22 participants (12% of 191).

The 22 participants were 10 women and 12 men, predominantly White British/White Other (17 participants), with a mean age of 53 years (range 26–80 years) and a mean body mass index at baseline of 32.6 (range 25.2–45.1). At outcome (12 months), 11 had achieved clinically significant weight loss (5% or more of baseline weight) and 11 had not. The 22 participants did not significantly differ from the other 169 trial participants in the intervention arm for: age, baseline weight, waist or body mass index, or final weight loss, but were more likely to be male (12/22 compared to 42/169, Chi^2^(1) = 8.5, *p* < 0.01) and to complete more sessions (mean 10.9 compared to 7.4, t(158) = 3.5, p < 0.01).

### The weight loss intervention

The Camden Weight Loss programme was offered to adults with weight categorised as clinically overweight or obese in primary care practices in a research trial [25]. The trial aimed to develop a locally delivered weight loss intervention, in line with the National Health Service *Health Trainers Initiative* [26], drawing health advisors from local communities, who are trained to support people in adopting healthier lifestyles by using psychological techniques to promote behaviour change. These techniques include supporting others to: choose a behaviour to change, set ‘SMART’ goals, plan behaviour change, improve confidence, review behaviour change, and embed behaviour change into their lifestyle [26]. The intervention was devised as a multi-component programme to promote behaviour change in line with National Institute for Health and Care Excellence guidance [27] and based on behaviour change models (Social Cognitive Theory, Goal Setting, Systems Thinking) [28–30]. Baseline and final weight were measured by research staff. The results of the randomised controlled trial of 381 participants, which included the 191 participants in the intervention arm, are published elsewhere [25].

Six advisors with a background in health care or exercise were trained to deliver a structured one-to-one intervention. The recordings included five of the advisors: one nurse, two osteopaths and two qualified personal fitness trainers, one of whom also had training in nutrition (CYQ Central YMCA Qualification Level 3 Award). Each participant was allocated to one advisor and were scheduled to attend 14 sessions, lasting 30 min per session, over 12 months in a primary care setting. The session length was intended to enable more in-depth discussion of weight management than is possible in a standard National Health Service primary care consultation (10 min), whilst being delivered to participants in their local practice. Sessions 9, 10 and 11 straddled the intervention mid-point (6 months).

Advisors attended two days of training, including:

(i) the intervention design and rationale
(ii) effective behaviour change strategies and principles of motivational interviewing
(iii) simulated practice in setting weight loss goals, talking about weight and behaviour change and addressing difficult issues.

Advisors were given a detailed manual listing the goals and content of each session, including handouts for participants in some sessions, and a 20-page booklet on Helping People Change Behaviour, including worked examples of techniques for: motivational interviewing, agenda setting, assessing importance and confidence, listening and informing. During the intervention, advisors attended additional group meetings, including further training in motivational interviewing techniques, and met with research staff individually to discuss intervention progress and any issues in intervention delivery.

Each consultation included a review of progress, recording and reviewing pedometer counts, taking weight and waist measurements, reviewing weight loss progress, introducing a new topic, goal setting, making an action plan and confirming the next appointment. The review included discussing the participant’s experience and success with the previous session’s topic. The intervention schedule is shown in Table 1. Sessions were delivered on a regular schedule with tapering frequency: fortnightly for 12 weeks, 3-weekly to 27 weeks, 4-weekly to 35 weeks and a 12-week interval to the last session. Further details of the intervention are published elsewhere [25]. The topics addressed in sessions 9, 10 and 11 were: positive and negative thinking, responding to situations where you might ‘slip up’, social eating, and staying on course in the long term.

**Table 1:** CAMWEL weight loss programme sessions

Session	Week	Topic	Content
Pre-intervention: baseline measurements by researchers			
1	0	Getting started	Eliciting personal reasons for losing weight, commitment to programme
2	2	Changing habits	Importance of changing habits permanently
3	4	Healthy eating	Regular meals, portion sizes, easy food swaps
4	6	Let’s get active	Incorporating physical activity into daily lifestyle
5	8	Taking charge of your environment	Acting on environmental cues
6	10	Eating when out and about	Making healthy choices, discuss alcohol if appropriate
7	12	Tip the calorie balance	Energy balance equation, and action planning
8	15	Positive thinking	Ways to stop negative thoughts
9	18	Getting off the slippery slope	Slips, and getting back on course
10	21	Social eating	Difficult social settings and how to control eating
6 month measurements by researchers			
11	27	Staying on course	Identify successes, and how to stay on course
12	31	Staying active	Additional physical activity to be added to the routine
13	35	Managing stress	How stress can affect weight, how to overcome
14	47	Reshaping habits	Review progress, how to continue in long term
12 month measurements by researchers			

### Measures

#### Treatment fidelity

Checklists were devised for the three sessions by itemising the session content from the manual, using the same wording for items repeated across sessions. Eight topics were included in every session: (1) reviewing overall progress, (2) reviewing previous topic and handouts, (3) reviewing pedometer counts and physical activity, (4) taking weight and waist measurements, (5) reviewing weight change, (6) presenting new topic, (7) setting goals, making action plans and assigning home activities, (8) setting date of next appointment. Due to the detail in the manual, the initial checklists contained 41, 40, and 52 items respectively for the three sessions. The final checklist for session 9 is shown in Table 2 as an example.

**Table 2:** Example treatment fidelity checklist: session 9

Item	Topic
1. Reviewing overall progress	
1	Previously set goals or intentions are reviewed
2	Feedback on performance is given
3	General encouragement is given
2. Reviewing previous topic and handouts	
4	Positive Thinking handout is reviewed
	The following questions are taken into consideration when reviewing the Positive Thinking handout
5	• What negative thoughts did you catch yourself thinking?
6	• When was this (in what situation)?
7	• Were you able to stop them?
8	• Did you talk back with positive thoughts?
9	• How did positive thinking help with eating a healthy diet?
10	• What about changes to your activity habits?
11	• Did you manage to go for a 30-min walk every day?
12	Any barriers are uncovered
	Usefulness of the other handouts is reviewed:
13	• CAMWEL walks handout
14	• Steps/Walks Chart handout
15	• Rate Your Plate handout
3. Reviewing pedometer counts and physical activity	
16	Pedometer counts are recorded
17	Participants are asked if they wore the pedometers every day
18	(a) If the participant did not wear the pedometer every day, they are asked what got in the way OR (b) If the participant did wear the pedometer every day, they are praised
19	Efforts made by the participant are appreciated
20	Advisor is positive and non-judgmental; good things are noticed
4. Taking weight and waist measurements	
21	Waist circumference is measured
22	Weight is measured
5. Reviewing weight change	
23	(a) If participant has lost weight they are congratulated OR (b) If the participant has not lost weight they are helped to develop a plan to address their particular problem
6. Presenting new topic – Avoiding The Slippery Slope of Changing Habits for Life	
24	‘Slips’ are defined in a manner that is relevant to the participant
25	Advisor discusses what to do after a ‘slip’ to get the participant back on their feet again
26	Advisor helps to identify some things or situations when the participant ‘slips’ from healthy eating and being active
7. Setting goals, making action plans and assigning home activities	
27	Participant is encouraged to continue to get back on track
	The following leaflets are given:
28	• Camden Change4Health Walks
29	• Recipe book (British Heart Foundation ‘Healthy Meals, Healthy Hearts’ or ‘Food Should be Fun… And Healthy’)
30	Participant is reminded to continue to wear their pedometer and record their steps
8. Confirming date of next appointment	
31	The appointment for the next session is confirmed

The checklist was piloted using a scoring key of 0 (not done), 1 (partially done) and 2 (completely done) for most of the items (e.g. ‘feedback is given on performance’), with some simple items (e.g. ‘waist circumference is measured’) assessed on a binary scale of 0 (not done) and 1 (done). However, low frequency and brevity of advisor behaviours observed during piloting indicated that measurement was better suited to assessing the presence of behaviours in comparison to expected content, rather than a combination of presence and quality. The scoring key for all items was converted to a binary scale (done/not done). An additional category (‘not recorded’) noted where items could not be rated due to poor sound quality. A rater crib sheet specified item content, including strategies or examples the advisors had been encouraged to use.

#### Motivational interviewing

The Motivational Interviewing Treatment Integrity Scale [31] assesses adherence to and competence in using motivational interviewing, with good inter-rater reliability reported [32, 33]. Global ratings are made for five dimensions: Evocation, Collaboration, Autonomy/Support, Direction and Empathy on a 5-point scale, and a summary score: Spirit of Motivational Interviewing. Behaviour counts are made for seven aspects: Giving Information, Closed Questions, Open Questions, Simple Reflections, Complex Reflections, Motivational Interviewing Adherent Behaviours and Motivational Interviewing Non-adherent Behaviours. Further summary scores are also computed.

#### Therapeutic alliance

The 14-item Alliance scale of the Primary Care Therapy Process Rating Scale [34] assesses the quality of the professional-patient therapeutic bond in psychological interventions conducted in primary care settings. It was designed for research into treatment fidelity and process-outcome relationships and has good internal consistency (Cronbach’s alpha 0.88). Items are scored on a 7-point scale, with anchors at four points (not at all, somewhat, considerably, extensively).

### Rater training

Raters were blind to participants’ weight loss outcomes. The raters (LA and GB) practised using consultations not included in the analysis (five for treatment fidelity, 18 for motivational interviewing and 10 for therapeutic alliance) and discussed discrepancies. The raters independently rated consultations in batches of five and reconvened to discuss discrepancies. For motivational interviewing, a third rater (LN) independently rated six consultations and met with the raters to discuss discrepancies. For therapeutic alliance, a third rater (EG) independently rated three consultations and met with raters to discuss discrepancies and provide additional examples of ratings. Raters coded each consultation four times: once for treatment fidelity, twice for motivational interviewing (global ratings followed by behaviour counts) and once for therapeutic alliance.

## Results

### Treatment fidelity analysis

#### Inter-rater reliability

Overall and specific agreement, for positive and negative agreement, were calculated for each item [35, 36]. Overall agreement was 0.82 (i.e. for 770/937 decisions, the raters agreed that the item had been done or not done). Items with low inter-rater reliability (defined as agreement in less than 70% of decisions) or with too many missing (due to issues with sound in the recording) were excluded. The items deleted were: (1) initial items, as advisors did not necessarily start the recording immediately, (2) items that could not be reliably recorded from audio-only recordings, e.g. ‘advisor shows acceptance of the participant’ (which could have been communicated non-verbally) or ‘showing the participant’s weight change on a graph’, (3) repeated items (e.g. ‘reviewing previously set goals or intentions’ appeared in two places in the session plan), and (4) items that could not be assessed without knowledge of the participant’s perspective (e.g. ‘uses analogies that are meaningful to the participant’).

The resulting checklists contained 31, 32 and 35 items respectively for the three sessions. In the final versions, the proportion of overall agreement was 0.90 (0.88, 0.90 and 0.93 respectively). The proportions of specific agreement were 0.88 for positive agreement and 0.92 for negative agreement.

#### Fidelity identified from the recordings

Overall, a mean of 41% of scheduled content was addressed, with session totals of 39, 35 and 49% respectively (Table 3). The amount of content addressed per participant ranged from 24 to 54% (SD 10%).

**Table 3:** Percentage session content addressed by the advisors

Content of topics	Session 9	Session 10	Session 11	Total *N* = 22
	*N* = 10	*N* = 6	*N* = 6	
1. Review of general progress Discussing goals from previous session General feedback and encouragement Uncovering barriers	85%	71%	98%	85%
2. Review of previous topic and handouts Participant’s use of information from last session (Positive Thinking/Avoiding the Slippery Slope/Social Eating) Uncovering barriers Participant’s use of handouts	13%	0%	3%	8%
3. Review of pedometer counts and physical activity Pedometer use and step counts Appreciation of participant’s efforts Use of handout about walks	42%	60%	12%	39%
4. Taking weight and waist measurements	100%	100%	92%^a^	98%
5. Reviewing weight change	75%	83%	100%	84%
6. Presenting new topic Discussing topic with examples (Avoiding the Slippery Slope, Social Eating, Staying on Course)	68%	21%	79%	60%
7. Setting goals, developing action plans and assigning home activities Encouragement to keep on track New information leaflets Reminder to use pedometer	11%	13%	39%	24%
8. Setting date of next appointment	80%^a^	83%	100%	86%
Totals	39%	35%	49%	41%

^a^2 data points missing due to problems with the recording

Content included most frequently was: taking weight and waist measurements (98%), setting date of next appointment (86%), reviewing general progress (85%), and reviewing weight change (84%). The most frequent items were ‘giving encouragement’ and ‘showing appreciation of participant’s efforts’ (95% in reviewing general progress and 88% in reviewing pedometer counts and physical activity).

Topics with lower or more variable frequency were: reviewing participant’s use of the previous session’s topic and handouts (8%), reviewing pedometer use, reviewing step counts and physical activity (excluding the item about appreciation of participant’s efforts) (19%), setting goals, developing action plans and assigning home activities (24%), and presenting the new topic, which varied from 21% for session 10 (Social Eating) to 79% for session 11 (Staying on Course).

Mean consultation length was 18.9 min (SD 7.6, range 9.0–30.4). Quantity of content correlated with consultation length (Spearman’s rho 0.73, *p* < 0.01).

### Motivational interviewing

#### Inter-rater reliability

For the global dimensions, using the categories described by Cicchetti [37] the intra-class correlation coefficients (two-way random, testing for consistency) were excellent for Direction, fair for Empathy, and poor for Evocation, Collaboration, Autonomy/Support and Spirit of Motivational Interviewing (Table 4).

**Table 4:** Motivational interviewing mean scores by rater and inter-rater reliability for global dimensions and behaviour counts

Global dimensions	Rater 1 Mean (SD)	Rater 2 Mean (SD)	ICC(2,2)	95% CI Lower	95% CI Upper
Evocation	3.2 (1.0)	3.5 (0.7)	0.34	−0.58	0.73
Collaboration	3.3 (0.9)	3.2 (0.6)	0.37	−0.52	0.74
Autonomy/Support	3.1 (0.7)	3.4 (0.6)	Scale not reliable^a^		
Direction	4.2 (1.2)	3.8 (1.1)	0.83	0.59	0.93
Empathy	3.6 (0.7)	3.6 (0.6)	0.53	−0.14	0.80
Spirit of Motivational Interviewing	3.2 (0.8)	3.3 (0.5)	0.33	−0.62	0.72
Behaviour counts					
Giving Information	12.3 (5.5)	9.1 (4.1)	0.86	0.67	0.94
Closed Questions	3.0 (3.0)	2.3 (2.6)	0.65	0.16	0.85
Open Questions	2.0 (1.7)	2.4 (1.4)	0.53	−0.13	0.81
Simple Reflections	5.1 (3.9)	5.4 (4.4)	0.83	0.59	0.93
Complex Reflections	0.9 (1.2)	0.4 (1.0)	0.63	0.11	0.85
Motivational Interviewing Adherent	6.5 (4.3)	7.8 (5.1)	0.81	0.53	0.92
Motivational Interviewing Non-adherent	8.2 (6.9)	4.8 (3.5)	0.37	−0.52	0.74

^a^Landers [39]

For the behaviour counts, the intra-class correlation co-efficients showed excellent reliability for Giving Information, Simple Reflections, Complex Reflections and Motivational Interviewing Adherent, good reliability for Closed Questions, fair reliability for Open Questions and poor reliability for Motivational Interviewing Non-adherent.

#### Motivational interviewing identified from the recordings

The scale authors suggested that a mean score of 3.5 indicates ‘beginning proficiency’ and 4.0 indicates ‘competency’ for the global dimensions [31]. The mean scores for Evocation, Collaboration, Autonomy/Support and Spirit of Motivational Interviewing fell below the threshold for ‘beginning proficiency’, and Empathy was at ‘beginning proficiency’ (Table 5). The mean score for Direction was high, indicating that advisors maintained focus on the target topic of weight loss.

**Table 5:** Motivational interviewing summary scores for behaviour counts and thresholds for proficiency

Summary score	Mean (SD)	Thresholds for proficiency from Moyers et al. 2010	
		Beginning proficiency	Competency
Reflection to Question ratio	1.8% (2.3)	1%	2%
Percent Open Questions	54.6% (27.4)	50%	70%
Percent Complex Reflections	9.3% (9.2)	40%	50%
Percent Motivational Interviewing Adherent	53.1% (17.2)	90%	100%

Mean total questions asked by the advisors was 4.8 (SD 3.0) and mean total reflections was 5.9 (SD 4.5). Summary scores for the behaviour counts fell between the scale authors’ suggested scores for ‘beginning proficiency’ and ‘competency’ for Reflection to Question ratio and Percent Open Questions, and below the threshold for ‘beginning proficiency’ for Percent Complex Reflections and Percent MI-Adherent.

### Therapeutic alliance

#### Inter-rater reliability and internal consistency

Excellent internal consistency was found (Cronbach’s alpha 0.92). Intra-class correlation coefficients (Table 6) showed excellent reliability for two items (warmth and empathy), good reliability for four items (involvement, rapport, client self-discloses thoughts and feelings, and client and therapist agree on the kind of changes to make), fair reliability for five items (supportive encouragement, client expresses emotions, client works actively with therapist’s comments, client and therapist share same sense about how to proceed, and client and therapist agree on salient themes), and poor reliability for the remaining three items.

**Table 6:** Alliance mean scores by rater and inter-rater reliability

Scale items	Rater 1 Mean (SD)	Rater 2 Mean (SD)	ICC(2,2)	95% CI Lower	95% CI Upper
1. Supportive encouragement	4.9 (1.8)	5.1 (1.3)	0.58	−0.01	0.83
2. Convey expertise	4.1 (1.5)	4.5 (1.0)	Scale not reliable^a^		
3. Therapist’s communication style	4.4 (1.3)	4.6 (0.6)	0.07	−1.26	0.61
4. Involvement	4.8 (1.4)	4.8 (1.1)	0.73	0.34	0.89
5. Warmth	4.8 (1.4)	4.6 (1.3)	0.81	0.54	0.92
6. Rapport	4.3 (1.7)	4.6 (1.2)	0.73	0.35	0.89
7. Empathy	3.8 (1.6)	4.6 (1.3)	0.79	0.50	0.91
8. Client self-discloses thoughts and feelings	4.6 (2.1)	4.5 (1.3)	0.67	0.21	0.86
9. Client expresses emotions	2.9 (1.6)	2.6 (1.5)	0.56	−0.07	0.82
10. Client works actively with therapist’s comments	3.1 (1.6)	4.0 (1.0)	0.52	−0.17	0.80
11. Client shows confidence in therapy and therapist	3.7 (1.6)	4.0 (1.3)	0.36	−0.55	0.73
12. Client and therapist agree on the kind of changes to make	3.2 (1.9)	3.8 (1.4)	0.65	0.17	0.86
13. Client and therapist share same sense about how to proceed	3.6 (1.4)	3.5 (1.4)	0.51	−0.19	0.80
14. Client and therapist agree on salient themes	4.0 (1.2)	3.8 (0.9)	0.55	−0.09	0.81
Total score	4.0 (1.1)	4.2 (0.8)	0.72	0.33	0.89

^a^Landers [39]

#### Therapeutic alliance identified from the recordings

Mean total score was 4.1 (SD 0.8) indicating that the consultations were being rated around the mid-point, between the anchor points of ‘somewhat’ and ‘considerably’. Of the 11 items with fair to excellent reliability, items with a mean score above the mid-point were: supportive encouragement (mean 5.0, SD 1.3), involvement (mean 4.8, SD 1.1), warmth (mean 4.7, SD 1.2), client self-discloses thoughts and feelings (mean 4.5, SD 1.5), rapport (mean 4.4, SD 1.3) and empathy (mean 4.2, SD 1.3).

### Relationship of process measures to weight outcome

Using independent t-tests to compare the 11 participants who had lost 5% or more of their baseline weight with the 11 participants who had not, there was no difference between the groups for: (i) consultation length, (ii) percentage of total content covered, (iii) motivational interviewing: Motivational Interviewing Spirit, percentage open questions, percentage complex reflections, percentage Motivational Interviewing Adherent, (iv) therapeutic alliance total score (Table 7).

**Table 7:** Comparison of consultations by participants’ final weight change

Mean (SD) score	Lost 5% or more of baseline weight (N = 11)	Did not lose 5% or more of baseline weight (N = 11)	Statistic	Significance	95% CI Lower	95% CI Upper
Fidelity						
Consultation length (minutes)	21.7 (7.5)	16.1 (6.9)	t(20) = 1.8	*p* > 0.05	−0.81	11.98
Percentage total content	40.3 (15.1)	38.9 (10.6)	t(20) = 0.2	*p* > 0.05	−10.23	33.43
Motivational interviewing						
Motivational Interviewing Spirit	3.4 (0.4)	3.1 (0.6)	t(20) = 1.3	*p* > 0.05	−0.18	0.73
Percentage open questions	59.0 (30.3)	50.3 (24.7)	t(20) = 0.7	*p* > 0.05	−15.83	33.43
Percentage complex reflections	8.2 (9.5)	10.4 (9.1)	t(20) = −0.6	*p* > 0.05	−10.48	6.06
Percentage Motivational Interviewing Adherent	55.8 (15.6)	50.5 (19.0)	t(20) = 0.7	*p* > 0.05	−10.15	20.75
Therapeutic alliance						
Alliance total score	4.3 (0.7)	3.9 (1.0)	t(20) = 1.0	*p* > 0.05	−0.39	1.01

## Discussion

Intervention fidelity should be improved by providing advisors with training, supervision and supporting materials [21]. Nonetheless, observed adherence to intervention content was lower than expected. Paradoxically, having detailed session content may cause a conflict between achieving an intervention that can be *consistently* delivered and one that can be *realistically* delivered. The development of the fidelity measure revealed a relatively high number of items for the intended consultation length. However, advisors were not routinely using the full time allocation, with the average duration of the consultations being a third less than the time scheduled. This suggests that health advisors were selective in delivering content. Certain elements were performed consistently, such as reviewing general progress and taking weight and waist measurements, whilst others were performed inconsistently, such as reviewing participants’ use of information from the previous session and goal-setting. The findings indicated that advisors focused more on practical elements and education than on exploring participants’ perspectives. The latter is potentially more challenging, despite the availability of time and relationship continuity. Notwithstanding the detailed guide to content, differing levels of skill are required across intervention components. This may highlight a limitation of interventions designed to be delivered by trained advisors rather than by traditionally trained health professionals, in that elements of the intervention requiring more advanced psychological consultation techniques were not delivered, despite the availability of time.

The advisors knew they were being recorded, indeed, they switched on the recording equipment, as is common in UK primary care settings. During the five-month recording period, all consultations were recorded (whether or not at the mid-point of the intervention), to ‘normalise’ the routine of recording, of which a fifth (22/104) were analysed. The raters reported that the advisors appeared to have a ‘routine’ for the consultations and that the language used (for example, in beginning the consultation and initiating the ‘taking measurements’ task) indicated that this was a routine familiar to the participants. This suggests that the aim of capturing a well-developed relationship and consultation routine was achieved by recording at the intervention mid-point. Whilst it cannot be ruled out, there was no evidence to suggest that the advisors were behaving differently whilst being recorded.

Higher inter-rater reliabilities were achieved for ‘basic’ and specific skills in motivational interviewing (e.g. whether the advisor maintains a focus on the target topic, demonstrates an empathic approach, or asks closed questions). The authors of the scale noted that it performs better for rating ‘entry level’ than expert therapeutic behaviours, and specifically, for measuring empathy and micro-skills (such as using open and closed questions) rather than advanced skills (such as creating a discrepancy between client values and behaviours or eliciting change talk) [32]. The findings of the present study are consistent with this. It is, however, easier to code a behaviour reliably when it is present, as behaviours which appear to meet the criteria can be scrutinised and any discrepancies between raters discussed.

The findings suggested that the advisors were operating at ‘entry level’ proficiency in motivational interviewing, which is consistent with the advisors’ level of experience and skill. Advisors consistently demonstrated an empathic approach and maintained a focus on the topic of weight loss. This concurs with the results about fidelity, which also found the advisors to be consistently encouraging and supportive. However, more advanced therapeutic skills in motivational interviewing were not observed. This ‘layering’ of skills in motivational interviewing, with increasing complexity requiring considerable experience and training, is consistent with other research [24].

In terms of therapeutic alliance, higher inter-rater reliability was achieved for affective qualities of the relationship (e.g. involvement, warmth, rapport and empathy) than specific skills (e.g. client works actively with therapist’s comments). Consistent with the findings from the other two measures, advisors demonstrated ‘entry level’ proficiency, achieving higher ratings for aspects of the quality of the interpersonal climate, such as supportive encouragement, involvement, warmth, rapport and empathy.

Overall, the findings demonstrated that certain aspects of the intervention were consistently delivered. Participants’ weight was checked, information was provided, and sessions maintained a focus on the target outcome. Furthermore, these tasks were conducted in the context of a warm and supportive advisor-participant relationship. Interviews with participants in the Camden Weight Loss trial reported elsewhere revealed that the most valued aspects of the intervention were the relationship they formed with the advisor, followed by regularity of meetings [38].

The aim of the trial was to examine the feasibility and effectiveness of a weight management programme which was centrally organised but locally delivered in a primary care setting. Other research examining consultations in primary care in which weight management is discussed has demonstrated the importance of training health professionals in weight management interventions [18]. The results of the present study, however, highlight the importance of ongoing training and supervision. Multi-component weight management interventions comprise a spectrum of tasks and skills at varying levels of sophistication, which take time to acquire and develop.

An important determinant of intervention delivery is the congruence between the aims of the intervention and the experience and skill of the provider. One solution may be to use recorded consultations during supervision to provide feedback about fidelity and discuss strategies the advisor might use to achieve the intervention aims. In addition to providing ongoing training and supervision, another solution might be to alter the complexity of the intervention over time, as advisors’ experience and skill increase.

This study had several limitations. Consultation recordings were not available for all participants in the study, as they were gathered during a five-month period and participants varied in their start date for the 12-month intervention. Recordings were also subject to the vagaries of the primary care settings, including technical failure. The present sample attended a greater number of sessions compared to others in the intervention group, as those included necessarily continued to at least session 9, and were more likely to be male, although there is no clear explanation for this (there was no gender difference in the number of sessions completed). The small sample size made it difficult to assess the impact of variation in fidelity on intervention outcome (final weight change). Nonetheless, the observed consultations appeared to provide a representative picture of the intervention as delivered in practice.

The health advisors in the study were recruited and trained as recommended by the National Health Service *Health Trainers Initiative* [26]. However, the study did not examine delivery of the intervention by other types of advisors, such as professionals trained in primary care or psychological interventions. Advisors with different backgrounds and experience may have delivered the intervention differently.

## Conclusions

The results of randomised controlled trials and the effectiveness of complex interventions addressing behaviour change are dependent on the fidelity of the intervention delivered. Obesity statistics indicate that weight management interventions will continue to be required for the foreseeable future, emphasising the need for effective intervention delivery. This study has demonstrated that an independent process evaluation can identify the components of a complex intervention which are and are not reliably delivered. As these interventions are complex and layered, advisors delivering such interventions require considerable support, training and ongoing supervision to support those attempting to achieve significant weight loss.

## Abbreviations

CAMWEL: Camden Weight Loss
CI: confidence interval
EG: Emma Godfrey
GB: Gabriella Baez
ICC: intra-class correlation coefficient
KN: Kiran Nanchahal
LA: Liane Al-Baba
LN: Lorraine Noble
N: number
NHS: National Health Service
NICE: National Institute for Health and Care Excellence
NT: Nicki Thorogood
SD: standard deviation
UCL: University College London
UK: United Kingdom
